# Comparative analysis of body mass index and obesity-related anthropometric indices for mortality prediction: a study of the Namwon and Dong-gu cohort in Korea

**DOI:** 10.4178/epih.e2024066

**Published:** 2024-07-17

**Authors:** Ye Rim Kim, Min-Ho Shin, Young-Hoon Lee, Seong-Woo Choi, Hae-Sung Nam, Jeong-Ho Yang, Sun-Seog Kweon

**Affiliations:** 1 Interdisciplinary Program of Public Health, Chonnam National University, Hwasun, Korea; 2 Department of Preventive Medicine, Chonnam National University Medical School, Hwasun, Korea; 3 Department of Preventive Medicine & Institute of Wonkwang Medical Science, Wonkwang University College of Medicine, Iksan, Korea; 4 Department of Preventive Medicine, Chosun University College of Medicine, Gwangju, Korea; 5 Department of Preventive Medicine, Chungnam National University College of Medicine, Daejeon, Korea; 6 Gwangju-Jeonnam Regional Cancer Center, Chonnam National University Hwasun Hospital, Hwasun, Korea

**Keywords:** Obesity, Weights and measures, Mortality

## Abstract

**OBJECTIVES:**

This study investigated the associations between several obesity-related anthropometric indices and mortality in middle-aged and elderly populations to compare the indices’ predictive ability with that of the body mass index (BMI).

**METHODS:**

We analyzed data on 12 indices calculated from 19,805 community-based cohort participants (average age, 63.27 years; median follow-up, 13.49 years). Each index was calculated using directly measured values of height, weight, waist circumference (WC), and hip circumference (HC). We calculated hazard ratios (HRs) and 95% confidence intervals (CIs) for each index using Cox regression and evaluated mortality prediction with the Harrell concordance index (c-index).

**RESULTS:**

Adding anthropometric indices to the basic mortality model (c-index, 0.7723; 95% CI, 0.7647 to 0.7799) significantly increased the predictive power of BMI (c-index, 0.7735; 95% CI, 0.7659 to 0.7811), a body shape index (ABSI; c-index, 0.7735; 95% CI, 0.7659 to 0.7810), weight-adjusted waist index (WWI; c-index, 0.7731; 95% CI, 0.7656 to 0.7807), and waist to hip index (WHI; c-index, 0.7733; 95% CI, 0.7657 to 0.7809). The differences between the BMI model and the other 3 models were not statistically significant.

**CONCLUSIONS:**

In predicting all-cause mortality, the ABSI, WWI, and WHI models based on WC or HC had stronger predictive power than conventional risk factors but were not significantly different from the BMI model.

## GRAPHICAL ABSTRACT

[Fig f2-epih-46-e2024066]

## Key Message

• Several obesity indices provided predictive value for all-cause mortality but were not superior to body mass index

• Obesity indices that take abdominal circumference into account are likely to be more useful for predicting mortality.

## INTRODUCTION

The World Health Organization has published a factsheet indicating that in 2022, the global adult population aged > 18 years and defined as overweight surpassed 2.5 billion individuals, of which more than 890 million were classified as obese [1]. The global prevalence of obesity has more than doubled since 1990, with an ongoing upward trend [1]. Obesity is recognized as a significant risk factor that contributes to the development of various diseases, including cardiovascular disease (CVD), cancer, diabetes, and chronic kidney disease [2]. These health risks of obesity not only increase mortality, but also incur a substantial economic burden [3,4]. The Global Burden of Disease study reported that in 2017, approximately 4.7 million deaths were attributable to obesity, positioning it as the fourth leading cause of mortality worldwide [5].

Obesity is typically quantified using the body mass index (BMI), which has been observed to exhibit a J-shaped or U-shaped correlation with overall mortality in many studies worldwide [4,6]. The ease of measuring and categorizing obesity through BMI has made it a commonly used tool for classifying individuals as obese or not and for monitoring the obesity epidemic at the population level. Despite its advantages, BMI presents certain limitations; in particular, the fact that it is calculated solely based on height and weight leads to an inability to differentiate between body fat and lean mass or assess fat distribution [7,8]. Consequently, BMI is deemed a specific yet insensitive measure for identifying individuals with excess body fat [9]; as such, it offers a somewhat crude approach to capturing the spectrum of metabolic health conditions associated with obesity. BMI’s representation of body fat variance is reported at merely 25% [10], highlighting its inadequacy in accurately diagnosing sarcopenic obesity—a condition characterized by a significant reduction in lean mass relative to fat mass—in older adults [9,11]. Moreover, individuals with above-average muscle mass, such as muscular athletes, may be miscategorized as overweight or obese [12]. Given the stronger association of visceral fat with obesity-related diseases compared to subcutaneous fat, considering fat distribution is crucial for an accurate assessment of obesity risk [13]. However, the inherent anatomical limitations of BMI preclude it from providing information on body fat distribution [9].

Recent developments in anthropometric indices have incorporated waist circumference (WC) and hip circumference (HC) as key clinical proxies for indirect assessment of abdominal adiposity, thus serving as predictors of morbidity and mortality risks [14,15]. Several studies have indicated that adiposity measures, such as the waist-to-hip ratio (WHR) and waist-to-height ratio (WHtR), surpass BMI in predicting morbidity and mortality [15-17]. The dependence of WC on body size-related factors, such as height [18], has prompted the development of various obesity-related anthropometric indices to assess adiposity more accurately, validate associations with morbidity or mortality, and compare diagnostic or predictive performances. However, most of these new indices have been developed and validated primarily on populations in Europe or the United States [19-23], with limited evidence available for other cultures [14,24]. The gap is particularly relevant for Asian populations, which typically exhibit lower BMI and body fat levels than other ethnic groups [25,26], underscoring the need for further evidence to validate the applicability of Western-developed anthropometric indices in these populations.

This study aimed to evaluate the mortality-predictive ability of several anthropometric indices calculated using WC and to compare their predictive ability with that of conventional risk factors and BMI within Korean community-based prospective cohorts.

## MATERIALS AND METHODS

### Study subjects

This study used data focusing on Korean community residents from the Namwon Study (n=10,667; 2004-2007) and the Donggu Study (n=9,260; 2007-2010). The Namwon Study targeted individuals aged 45 years to 74 years who resided in Namwon, a rural municipal city in Jeonbuk Province, while the Dong-gu Study focused on those aged 50 years or older who lived in Dong District, Gwangju Metropolitan City. Detailed information regarding the study participants and measurement methods can be found in an earlier protocol paper [27]. Individuals who lacked data on height, weight, WC, HC, or death date were excluded from the analysis (n=122). Consequently, the remaining 19,805 individuals were included in the analysis.

### Covariates

The participants’ demographic factors, health behaviors, and medical histories were assessed via structured questionnaires. Educational levels were categorized as follows: no education or elementary school graduate, middle school or high school graduate, and university graduate or higher. Smoking status was classified into 3 groups: never, former, and current smokers. Diabetes was defined as taking anti-diabetic medication or having a fasting glucose level of ≥ 126 mg/dL. Hypertension was defined as taking anti-hypertensive medication or having a mean systolic blood pressure of ≥140 mmHg or diastolic blood pressure of ≥90 mmHg. A history of myocardial infarction, stroke, coronary heart disease, or cancer was ascertained through self-reporting.

### Anthropometric indices

Height was measured to the nearest 0.1 cm while the participants were barefoot, weight was measured to the nearest 0.1 kg while the participants wore light clothing without shoes, and HC and WC were measured to the nearest 0.1 cm above the light clothing. These measurements were converted to correspond to the formulas for each index. The indices and formulas employed in the analysis are presented below, with “Wt” denoting “weight” and “Ht” denoting “height.”

Body mass index (BMI)=Wt/Ht^2^

A body shape index (ABSI)=1,000*WC*Wt^–2/3^*Ht^5/6^ [19]

Abdominal volume index (AVI)=(2*(WC*100)^2^+0.7*(WC*100−HC*100)^2^)/1,000 [20]

Body roundness index (BRI)=364.2–365.5*(1−((0.5*WC/π)^2^/(0.5*Ht)^2^))^0.5^ [21]

Conicity index (ConI)=WC/(0.109*(Wt/Ht)^0.5^) [28]

Estimated total body fat (eTBF)=100*(–Z+A−B)/C, where A=(4.15*WC*39.3701), B=(0.082*Wt*2.20462), C=(Wt*2.20462), Z=98.42 [male] or Z=76.76 [female] [29, 30]

Relative fat mass (RFM)=64−(20*Ht/WC)+(12*S), where S=0 [male] or S=1 [female] [31]

Hip index (HI)=(HC*100) *(Wt/< Wt>)^–0.482^*((Ht*100)/< Ht>)^0.310^; here, < Wt> and < Ht> are the mean values for each sex, respectively [22]

Waist-to-hip ratio (WHR)=WC/HC

Waist-to-height ratio (WHtR)=WC/Ht

Weight-adjusted waist index (WWI)=(WC*100)/Wt^0.5^ [32]

Waist-to-hip index (WHI)=WHR*Wt^−1∕4^*(Ht*100)^1∕4^ [23]

### Ascertainment of death

Follow-up of the participants’ mortality was performed by linking database records from the national death registry, National Statistical Office, Korea. The date of death was followed until December 31, 2020, and the median follow-up period was 13.49 years (range, 0.30 to 16.98).

Censoring was set appropriately as follows: subjects who were still alive as of December 31, 2020, were censored at that date. Subjects who died during the follow-up period were considered to have experienced the event (death). Additionally, 325 accidental deaths corresponding to codes S00-T98, based on the 10th revision of the International Statistical Classification of Diseases and Related Health Problems, were separated from disease deaths so that competing survival analyses could be conducted.

### Statistical analysis

The t-test or chi-square test was conducted to compare the basic characteristics of the survivors and the deceased individuals. A survival analysis was carried out to assess the effects of anthropometric indices on overall mortality. Hazard ratios (HRs) and 95% confidence intervals (CIs) were calculated for each anthropometric index using the Cox proportional hazard model and Fine-Gray competing risk regression model, with adjustments for the following as covariates: age, sex, smoking status, educational level, hypertension, diabetes, and a history of severe chronic disease, including myocardial infarction, stroke, coronary heart disease, or cancer. A non-linear model of each HR was also established using restricted cubic splines in the Fine-Gray regression model. The non-linearity of the HRs of all anthropometric indices, including BMI, was tested using analysis of variance to compare the fit of the linear and non-linear models.

Harrell’s concordance index (c-index) was used to assess each anthropometric index’s predictive ability regarding mortality [33]. The basic prediction model of the c-index included age, sex, smoking status, educational level, hypertension, diabetes, and a history of severe chronic disease. The c-index of each anthropometric index was calculated by adding it to the basic model, and the significance of the difference between the c-index of each anthropometric index and the basic model (with BMI added to the basic model as a covariate) was evaluated. Anthropometric indices with significantly higher predictive ability than the basic model were stratified by the presence of chronic diseases, including hypertension, diabetes, or a history of severe chronic disease. To improve the comparison between decedents and survivors, an additional analysis using 1:1 propensity score matching (PSM) was performed between 3,220 decedents and 3,220 survivors for age, sex, smoking status, hypertension, diabetes, and a history of severe chronic disease. All data analysis was undertaken using R version 4.2.1 (R Foundation for Statistical Computing, Vienna, Austria) with the packages *survival*, *CsChange*, *rcs*, and *MatchIt*. Statistical significance was defined as a 2-sided p-value < 0.05.

### Ethics statement

All study participants provided informed consent, and the cohort studies received approval from the Chonnam National University Hospital Institutional Review Board (IRB No. I-2008-05-056, I-2014-215).

## RESULTS

Table 1 summarizes the baseline characteristics of the study participants. A higher frequency of deaths was observed among males, those with lower educational levels, smokers, individuals with hypertension or diabetes, and those with a history of severe chronic disease, including myocardial infarction, stroke, coronary heart disease, or cancer (Table 1).

Table 2 presents the mean of the anthropometric indices at the baseline survey. Significant differences were noted between survivors and deceased participants. The latter had higher mean values of height, ABSI, ConI, WHR, WWI, and WHI, whereas lower mean values were observed for weight, WC, HC, BMI, AVI, BRI, eTBF, RFM, HI, and WHtR (Table 2).

**Figure 1.**
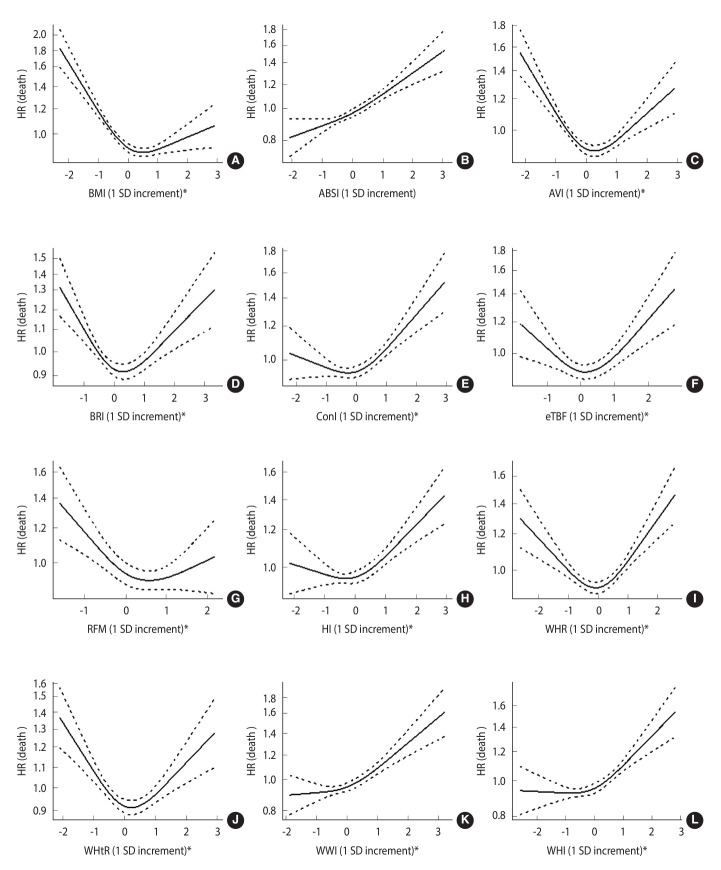
All-cause mortality HRs and 95% CIs for anthropometric indices. (A) Body mass index (BMI), (B) a body shape index (ABSI), (C) abdominal volume index (AVI), (D) body roundness index (BRI), (E) conicity index (ConI), (F) estimated total body fat (eTBF), (G) relative fat mass (RFM), (H) hip index (HI), (I) waist to hip ratio (WHR), (J) waist to height ratio (WHtR), (K) weight-adjusted waist index (WWI), and (L) waist to hip index (WHI). HR, hazard ratio; CI, confidence interval; SD, standard deviation. ^*^p for non-linear test <0.05.

Figure 1 depicts each anthropometric index’s HR for an increase of 1 standard deviation (SD) in all-cause mortality. In the restricted cubic spline regression model, only the association between ABSI and the risk of death was linear (non-linear p-value=0.270) (Figure 1).

Table 3 presents the HRs for non-accidental deaths from the competing risk analysis. In both the univariate and multivariate analyses, age, sex, smoking status, educational level, hypertension, diabetes, and a history of severe chronic disease were identified as significant risk factors for mortality (Table 3).

Table 4 shows the c-index values after anthropometric indices for all-cause mortality were incorporated. Integrating anthropometric indices into the basic model for mortality prediction led to higher c-index values for the models based respectively on BMI (c-index, 0.7735; 95% CI, 0.7659 to 0.7811), ABSI (c-index, 0.7735; 95% CI, 0.7659 to 0.7810), WWI (c-index, 0.7731; 95% CI, 0.7656 to 0.7807), and WHI (c-index, 0.7733; 95% CI, 0.7657 to 0.7809), which indicated an improvement in mortality predictive power compared to the basic model. ABSI, WWI, and WHI exhibited c-index values comparable to or lower than the BMI value, yet no statistically significant difference in mortality prediction was observed compared to the BMI model. Conversely, AVI, BRI, RFM, and HI displayed significantly inferior predictive power compared to BMI (Table 4). Additional PSM analysis showed almost the same results, except that the overall c-index values were lower than in the original model. The c-indices of BMI, ABSI, WWI, and WHI were 0.5625 (95% CI, 0.5518 to 0.5732), 0.5663 (95% CI, 0.5557 to 0.5769), 0.5614 (95% CI, 0.5508 to 0.5721), and 0.5634 (95% CI, 0.5528 to 0.574), respectively (data not shown).

Additionally, stratification analyses were conducted by categorizing the study population into individuals with and without chronic diseases (hypertension, diabetes mellitus, myocardial infarction, stroke, coronary heart disease, and cancer). These analyses indicated that when compared to the basic model, BMI, ABSI, WWI, and WHI had better predictive power regarding mortality among the participants with chronic diseases, but not among the healthy population (Table 5).

## DISCUSSION

This study evaluated the associations between obesity-related anthropometric indices and mortality within Korean community-based cohorts and compared each anthropometric index’s predictive ability regarding mortality. The addition of anthropometric indices to the basic mortality prediction model improved the predictive performance of 4 indices of the 12 indices (BMI, ABSI, WWI, and WHI). Notably, the indices reflecting WC and HC (ABSI, WWI, and WHI) had c-index statistics comparable to or lower than BMI, although these differences were not statistically significant.

Traditionally, BMI has been widely used as a metric for assessing and classifying obesity because of its simplicity, non-invasiveness, and affordability [9]. However, BMI has inherent limitations, including its inability to differentiate between body composition components, such as skeletal muscle mass and fat distribution, and its lack of consistency in cut-off values across different races, ages, and levels of muscularity, which have resulted in misclassifying individuals as obese [12,26,34].

To address the limitations of BMI, novel composite indicators incorporating WC have been proposed as alternative obesity-related anthropometric indices. Extensive research has been undertaken to investigate whether these indices outperform BMI in assessing obesity and predicting morbidity or mortality. A meta-analysis of 38 studies showed that a high ABSI was associated with higher risks of hypertension, type 2 diabetes, and CVD, and that ABSI served as a more effective predictor of all-cause mortality than BMI [24]. The Rotterdam Study and the European Prospective Investigation into Cancer and Nutrition (EPIC) study corroborated these findings, showing that ABSI exhibited a stronger association with overall mortality than BMI. However, these studies did not observe a statistically significant increase in c-statistics over the basic mortality prediction model [35,36]. In East Asian populations, ABSI has demonstrated a positive linear association with all-cause mortality [37,38] and comparable predictive power to BMI [32]. Nonetheless, according to the National Health and Nutrition Examination Survey, ABSI predicted mortality for individuals of White and African American heritage but not for those who identified as Hispanic [19].

Similarly, studies on WWI have revealed a positive association with hypertension, heart failure, and mortality [39,40], with some reports indicating improved predictive performance over BMI and ABSI [32,41]. WWI was developed in 2018 using a Korean cohort [32], and studies have used WWI for mortality prediction in both East Asian [41] and Western populations [42]. However, since WHI is a relatively new metric, there has been insufficient research on its association with mortality. Nonetheless, its positive correlation with certain cancer types has been observed in preliminary studies [23].

The primary distinction among these 3 anthropometric indices lies in whether they incorporate WC or HC, thereby reflecting central obesity levels. The WC measurement is widely accepted for estimating visceral adipose tissue (VAT) and abdominal subcutaneous adipose tissue (SAT), while HC is strongly correlated to abdominal SAT [43]. VAT, which is characterized by higher inflammatory and immune cell content [13], poses greater health risks than SAT and is closely associated with obesity-related morbidity and mortality [44,45]. In a recent meta-analysis of 72 cohort studies, traditional anthropometric indices including WC, WHR, and WHtR showed a significantly positive association with the risk of all-cause mortality [46]. Therefore, considering that WC independently predicts VAT irrespective of BMI or HC, whereas BMI loses its association with VAT or even shows an inverse relationship when adjusted for WC [47], the inclusion of WC in anthropometric indices seems crucial for accurately predicting obesity-induced morbidity and mortality.

Moreover, the associations between anthropometric indices and mortality vary by age, sex, and race. Sex differences in adipose tissue distribution influence the predictive performance of these indices [34], with males exhibiting a higher risk of CVD events associated with the distribution of android adipose tissue [48,49], while females risk increases after menopause due to hormonal changes [48]. Additionally, racial disparities in the predictive power of anthropometric indices for mortality underscore the importance of cross-racial validation studies to enhance generalizability. In the United States population, several studies have indicated that the associations of BMI [19,49,50], ABSI, and WC [19] with mortality vary by race or sex. The potential for racial differences in each anthropometric index’s predictive power for mortality persists. Hence, using a diverse population to evaluate age-specific, sex-specific, and race-specific associations between anthropometric indices and mortality is necessary. In this study, we assessed the associations between mortality and 12 obesity-related anthropometric indices, derived from height, weight, WC, and HC, in a Korean middle-aged and elderly population. The findings revealed that ABSI, WWI, and WHI exhibited predictive power comparable to or lower than BMI, although the differences were not statistically significant.

Despite the strengths of this study, including the use of urban and rural community-based cohorts, relatively long follow-up periods, and a sufficient number of deaths (n=3,394), several limitations should be acknowledged. First, the analysis considered only baseline anthropometric measurements and did not adjust for potential changes over time. Second, the study was conducted primarily with older adults, which limited the generalizability of the findings to younger age groups. Lastly, reverse causality could not be considered due to a lack of information on recent or pre-death weight changes among the study participants.

In conclusion, ABSI, WWI, and WHI demonstrated stronger predictive power for all-cause mortality yet did not significantly differ from BMI. Further research is warranted to explore racial and age-specific associations between anthropometric indices and mortality, particularly in East Asian populations, where evidence remains relatively scarce.

## Figures and Tables

**Figure f2-epih-46-e2024066:**
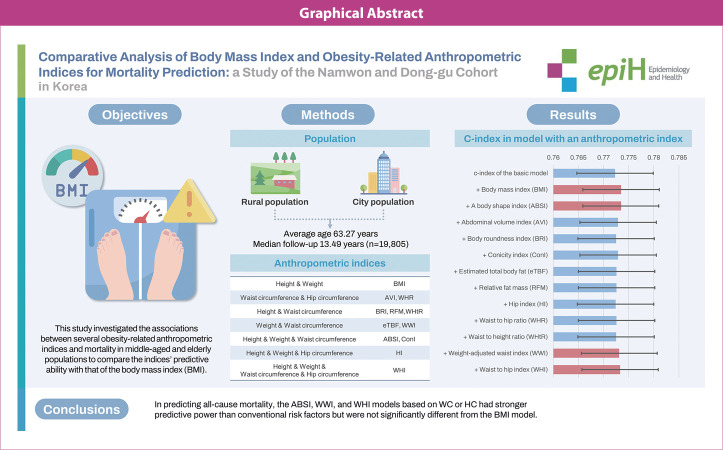


**Table 1.** Baseline characteristics of participants (n=19,805)

Characteristics	Survivors (n=16,107)	Deceased (n=3,698)	p-value^1^
Sex			<0.001
Male	5,707 (35.4)	2,143 (58.0)	
Female	10,400 (64.6)	1,555 (42.0)	
Age (yr)	61.9±7.7	69.0±7.6	<0.001
Follow-up (yr)	13.6±1.9	8.3±4.1	<0.001
Educational			<0.001
Lower than middle school	9,379 (58.2)	2,517 (68.1)	
Middle or high school	5,339 (33.1)	901 (24.4)	
University or higher	1,389 (8.6)	280 (7.6)	
Smoking status			<0.001
Never	11,455 (71.1)	1,778 (48.1)	
Former	2,823 (17.5)	1,129 (30.5)	
Current	1,829 (11.4)	791 (21.4)	
Hypertension^2^	6,488 (40.3)	1,825 (49.4)	<0.001
Diabetes mellitus^3^	2,447 (15.2)	936 (25.3)	<0.001
History of disease			
Myocardial infarction	103 (0.6)	52 (1.4)	<0.001
Stroke	483 (3.0)	276 (7.5)	<0.001
Coronary heart disease	578 (3.6)	226 (6.1)	<0.001
Cancer	517 (3.2)	293 (7.9)	<0.001

Values are presented as number (%) or mean±standard deviation.

1 By chi-square test or t-test as appropriate.

2 Hypertension included those who were taking anti-hypertensive medication or who had a mean systolic blood pressure of ≥140 mmHg or diastolic blood pressure of ≥90 mmHg.

3 Diabetes included those who were taking anti-diabetic medication or who had a fasting glucose level of ≥126 mg/dL.

**Table 2.** Baseline anthropometric indices of participants (n=19,805)

Variables	Survivors (n=16,107)	Deceased (n=3,698)	p-value^1^
Height (cm)	157.3±8.3	157.9±9.1	0.003
Weight (kg)	60.8±9.5	59.2±10.1	<0.001
Waist circumference (cm)	87.1±8.3	86.3±9.4	<0.001
Hip circumference (cm)	92.7±5.5	91.0±6.0	<0.001
Body mass index	24.5±2.9	23.7±3.2	<0.001
A body shape index	82.5±4.9	83.5±5.1	<0.001
Abdominal volume index	15.4±2.9	15.1±3.2	<0.001
Body roundness index	4.51±1.24	4.41±1.41	<0.001
Conicity index	1.28±0.08	1.29±0.08	<0.001
Estimated total body fat	35.5±13.0	32.3±15.1	<0.001
Relative fat mass	35.3±8.1	32.2±9.0	<0.001
Hip index	100.0±3.8	99.7±4.3	0.001
Waist to hip ratio	0.93±0.06	0.94±0.06	<0.001
Waist to height ratio	0.55±0.05	0.54±0.06	<0.001
Weight-adjusted waist index	11.21±0.81	11.28±0.90	<0.001
Waist to hip index	4.22±0.25	4.30±0.26	<0.001

Values are presented as mean±standard deviation.

1 By t-test.

**Table 3.** Hazard ratios of non-accidental deaths using the Fine-Gray competing risk regression model

Variables	Univariate	Multivariate
Age	1.13 (1.12, 1.13)	1.12 (1.12, 1.13)
Female	0.70 (0.55, 0.89)	0.58 (0.52, 0.64)
Smoking status		
Never	1.00 (reference)	1.00 (reference)
Former	2.31 (2.14, 2.50)	1.27 (1.14, 1.41)
Current	2.36 (2.16, 2.57)	1.76 (1.57, 1.97)
Education level		
Lower than middle school	1.00 (reference)	1.00 (reference)
Middle or high school	0.77 (0.71, 0.83)	0.79 (0.72, 0.853)
University or higher	1.01 (0.89, 1.15)	0.62 (0.54, 0.71)
Hypertension	1.47 (1.37, 1.57)	1.13 (1.05, 1.21)
Diabetes	1.93 (1.78, 2.08)	1.54 (1.42, 1.67)
History of disease		
Yes	1.00 (reference)	1.00 (reference)
Cancer	2.63 (2.32, 2.98)	1.95 (1.70, 2.23)
Coronary heart disease	1.80 (1.56, 2.07)	1.12 (0.80, 1.56)
Stroke	2.42 (2.13, 2.74)	1.26 (1.07, 1.47)
Myocardial infarction	2.34 (1.74, 3.15)	1.73 (1.53, 1.97)

Values are presented hazard ratio (95% confidence interval).

**Table 4.** All-cause mortality c-index values after adding each anthropometric index to a prediction model including conventional risk factors

Variables	c-index in model with an anthropometric index (95% CI)	p-value for comparison with the basic model	p-value for comparison with the BMI model
c-index of the basic model	0.772 (0.7647, 0.7799)	Reference^1^	-
+ BMI	0.7735 (0.7659, 0.7811)	0.018	Reference^1^
+ A body shape index	0.7735 (0.7659, 0.7810)	0.005	0.971
+ Abdominal volume index	0.7729 (0.7653, 0.7805)	0.115	0.142
+ Body roundness index	0.7725 (0.7649, 0.7801)	0.432	0.021
+ Conicity index	0.7729 (0.7653, 0.7805)	0.069	0.337
+ Estimated total body fat	0.7726 (0.7650, 0.7802)	0.222	0.085
+ Relative fat mass	0.7726 (0.7650, 0.7802)	0.338	0.009
+ Hip index	0.7724 (0.7648, 0.7800)	0.598	0.029
+ Waist to hip ratio	0.7726 (0.7650, 0.7802)	0.218	0.088
+ Waist to height ratio	0.7725 (0.7649, 0.7801)	0.397	0.016
+ Weight-adjusted waist index	0.7731 (0.7656, 0.7807)	0.013	0.585
+ Waist to hip index	0.7733 (0.7657, 0.7809)	0.005	0.734

c-index, concordance index; CI, confidence interval; BMI, body mass index.

1 Reference model included for age, sex, smoking status, educational level, hypertension, diabetes mellitus, and a history of myocardial infarction, stroke, chronic heart disease, or cancer.

**Table 5.** All-cause mortality c-index values after adding each anthropometric index to a model including conventional risk factors, stratified by the presence or absence of chronic diseases

Variables	c-index in model with an anthropometric index (95% CI)	p-value for comparison with the basic model	p-value for comparison with the BMI model
With chronic diseases (n=10,575, deaths=2,440)			
c-index of the basic model	0.7525 (0.7427, 0.7622)	Reference^1^	-
+ BMI	0.7544 (0.7447, 0.7641)	0.011	Reference^1^
+ A body shape index	0.7547 (0.7451, 0.7644)	0.002	0.753
+ Weight-adjusted waist index	0.7539 (0.7442, 0.7635)	0.023	0.601
+ Waist-hip index	0.7544 (0.7448, 0.7641)	0.002	0.986
Without chronic diseases (n=9,230, deaths=1,258)			
c-index of the basic model	0.7772 (0.7641, 0.7904)	Reference^1^	-
+ BMI	0.7776 (0.7644, 0.7908)	0.542	Reference^1^
+ A body shape index	0.7773 (0.7641, 0.7905)	0.829	0.691
+ Weight-adjusted waist index	0.7774 (0.7642, 0.7906)	0.596	0.791
+ Waist-hip index	0.7773 (0.7642, 0.7905)	0.641	0.695

c-index, concordance index; CI, confidence interval; BMI, body mass index.

1 Reference model included for age, sex, smoking status, educational level, hypertension, diabetes mellitus, and a history of myocardial infarction, stroke, chronic heart disease, or cancer.
